# Interphase Engineering in Lignin-Containing Nanocellulose Composites from Tropical Biomass: Evidence-Weighted Comparative Framework, Product Windows, and Biorefinery Constraints

**DOI:** 10.3390/polym18101238

**Published:** 2026-05-19

**Authors:** José Roberto Vega-Baudrit, Mary Lopretti

**Affiliations:** 1 Laboratorio Nacional de Nanotecnología (LANOTEC), Centro Nacional de Alta Tecnología (CeNAT-CONARE), San José 10109, Costa Rica; 2 Escuela de Química, Universidad Nacional, Heredia 86-3000, Costa Rica; 3 Faculty of Sciences.Nuclear techniques in Biochemistry and Biotechnology Departament, Nuclear Research Center, UdelaR, Montevideo 11400, Uruguay; mlopretti@gmail.com

**Keywords:** lignin-containing nanocellulose composites, interphase engineering, tropical biomass, biocomposites, evidence weighting, product windows, biorefinery, design rules

## Abstract

Tropical lignocellulosic residues are increasingly relevant feedstocks for lignin-containing nanocellulose composites, but their performance cannot be predicted from botanical origin or bulk lignin percentage alone. This review defines the interface as the geometrical boundary between phases and the interphase as the finite, compositionally graded region in which lignin distribution, nanocellulose morphology, adsorbed water, and the surrounding matrix jointly govern stress transfer and mass transport. Using an evidence-weighted framework, the literature is organized into the following categories: residual-lignin nanofibrils, redeposited-lignin systems, lignin nanoparticle assemblies, compatibilized thermoplastic hybrids, and all-lignocellulosic sheets. Representative quantitative observations show that controlled residual lignin can the increase water contact angle from approximately 35 degrees to 78 degrees and reduce oxygen permeability by up to 200-fold in nanopapers, while selected PLA/LCNF systems show tensile-strength and modulus increases of 37% and 61%, respectively; however, high or poorly distributed lignin can suppress fibrillation, lower viscosity, weaken gel networks, and reduce reproducibility. The most defensible near-term product windows are packaging layers, grease/oil barrier papers, coatings, paper-like multilayers, and selected porous media. Thermoplastic matrices remain process-sensitive, and biomedical, additive-manufacturing, nano-reactor, and energy-material claims require stronger validation of the extractables, rheology, humidity history, TEA/LCA metrics, and end-of-life behavior. This review, therefore, provides a critical, application-backward roadmap for tropical biorefineries in which interfacial function, wet handling, drying energy, and process integration are assessed together rather than treated as independent variables. The abbreviations used in the abstract are defined as follows: CNFs, cellulose nanofibrils; CNC, cellulose nanocrystals; LCNF, lignin-containing cellulose nanofibrils; LCNCs, lignin-containing cellulose nanocrystals; PLA, poly(lactic acid); PHB, polyhydroxybutyrate; PHAs, polyhydroxyalkanoates; PVA, poly(vinyl alcohol); DESs, deep eutectic solvents; TEA, techno-economic analysis; LCA, life-cycle assessment; ML, machine learning.

## 1. Introduction

The transition from fossil-derived polymers to renewable materials has made cellulose- and lignin-based systems central to sustainable polymer research and to emerging packaging, coating, pulp-and-paper, automotive, agricultural, and specialty-composite value chains. Global bio-based plastic capacity was reported at 2.31 million tonnes in 2025 and is projected to reach approximately 4.69 million tonnes by 2030, with packaging remaining the largest application segment; lignin-based biopolymer and resin markets are also expanding, although their commercial penetration remains constrained by process cost, standardization, and end-use qualification [1,2]. The relevant question is therefore no longer whether biomass can, in principle, replace petroleum, but whether biomass-derived building blocks can be engineered into interfaces and interphases that remain functional after fractionation, dewatering, drying, shaping, and service exposure. Nanocellulose is particularly important because its low density, high aspect ratio, large surface area, and network-forming capacity can provide barrier, mechanical, rheological, and surface functions in packaging, coatings, adsorption systems, and selected biomedical uses [3,4,5,6,7,8,9].

In parallel, lignin has moved from a pulping residue to a multifunctional aromatic macromolecule capable of UV screening, radical scavenging, surface energy modulation, char formation, and colloidal co-assembly. That reassessment is justified, but it also introduces a potential limitation: lignin is frequently discussed as a generic benefit rather than as a state-dependent interfacial variable whose value depends on where it resides, how it is distributed, and whether it survives processing in an interpretable form [10,11,12,13,14].

Recent reviews have already clarified broad preparation routes, generic property spaces, and application lists for lignin-containing nanocellulose and related cellulose–lignin hybrids [15,16,17,18,19]. A remaining unresolved issue is the lack of a sharper comparative framework that distinguishes non-equivalent architecture classes, links them to realistic product windows, and treats tropical residues as process-specific materials systems rather than as botanical labels.

This distinction matters because the strongest experimental studies do not support simple ‘more lignin is better’ or ‘less lignin is better’ narratives. Instead, they show that performance is governed more reliably by the lignin’s state, fibril morphology, moisture history, and shaping route than by bulk lignin fraction alone. Residual lignin can improve UV shielding, antioxidant behavior, rheology, wetting, and compatibility with less-polar phases, but coarse or redeposited lignin domains can suppress fibrillation, weaken network continuity, and destabilize films or composites [20,21,22,23,24].

The present review, therefore, repositions the field around interfacial/interphase engineering, explicit evidence weighting, and application-backward design. It does not treat tropical biomass as a generic substitute for wood, nor lignin-containing nanocellulose as an incompletely purified intermediate. Instead, it separates hybrid architecture classes, compares tropical residues as distinct process geographies, and asks which product windows remain scientifically and industrially credible once moisture management, drying burden, and biorefinery constraints are made explicit. The central hypothesis is condition-dependent: in tropical lignin–nanocellulose systems, the state of the lignin is usually more informative than its amount, but only when the architecture class and shaping route are specified.

Terminological note: In this review, interface denotes the geometrical boundary between two phases, whereas interphase denotes the finite, compositionally and structurally graded region near that boundary. In lignin-containing nanocellulose materials, that region may include cellulose surface charge, residual or redeposited lignin, adsorbed water, matrix chains, plasticizers, or coating additives. When the cited evidence only supports contact at the boundary, the manuscript uses interfacial language; when a finite gradient or processing-created zone is implicated, interphase language is retained.

**Figure 1:**
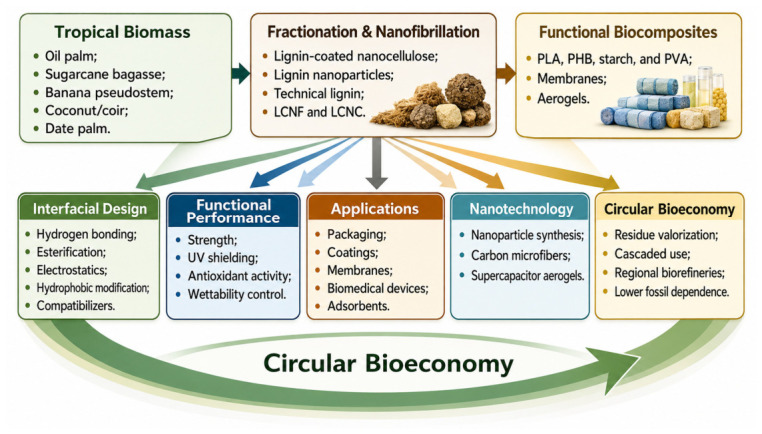
Evidence-weighted review logic from tropical residue family to interphase-enabled product window. Abbreviations: LCNFs, lignin-containing cellulose nanofibrils; LCNCs, lignin-containing cellulose nanocrystals; PLA, poly(lactic acid); PHB, polyhydroxybutyrate; PVA, poly(vinyl alcohol). Schematic illustration prepared for this review.

Figure 1 presents the review workflow as a five-step decision sequence: process geography, pretreatment severity, lignin state, shaping route, and product-window credibility. This framing avoids a linear ‘biomass → purification → application’ sequence and instead treats tropical residues as process-integration problems. In practical terms, the figure asks whether a specific residue stream can be converted into a stable, reproducible, and application-relevant interface before high-value claims are made.

## 2. Review Boundaries, Evidence Weighting, and Tropical Feedstocks

### 2.1. Review Boundaries, Architecture Classes, and Evidence Weighting

This is a structured comparative review, not a formal meta-analysis. The cited literature was organized into five recurring architecture classes: (i) residual-lignin CNFs/CNCs produced directly from partially delignified biomass, (ii) redeposited- or phase-separated lignin systems created during fractionation or drying, (iii) lignin nanoparticle or technical-lignin assemblies with nanocellulose, (iv) compatibilized or carrier-assisted thermoplastic hybrids, and (v) all-lignocellulosic sheets or molded structures in which lignin functions as an internal matrix or water-resistance phase [15,16,17,18,19].

Within each class, the evidence was compared using the same decision variables: feedstock and process geography, pretreatment severity, lignin state, nanocellulose morphology, shaping route, and application-relevant testing. Greater interpretive weight was assigned to studies that simultaneously report feedstock identity, lignin content and distribution, fibril or crystal morphology, surface chemistry or charge, moisture conditioning, and process history. Lower weight was assigned to studies that infer mechanisms from the lignin percentage alone, rely on severe drying or solvent exchange incompatible with plausible deployment, or claim interphase effects without reporting humidity and solids history.

This weighting logic matters because the field contains numerous qualitative trends yet still lacks directly comparable datasets. The present review, therefore, extracts robust tendencies and credible windows rather than universal thresholds. When the evidence is insufficiently normalized across humidity, drying, and the colloidal state, that limitation is stated explicitly rather than converted into a global design rule.

Under this framework, tropical biomass is treated as a mechanistic and logistical variable. The key question is not which crop is most suitable in isolation, but which residue family can reach a useful interface/interphase with the lowest combined burden of contamination control, wet handling, fractionation, dewatering, and shaping. The selected residues were therefore chosen because they represent contrasting tropical process geographies: mill-concentrated streams (oil palm and sugarcane), decentralized wet residues (pineapple and banana), and regionally abundant arid-zone residues (date palm). The selection is not exhaustive; it is intended to cover residue systems for which nanocellulose/lignin evidence and deployment constraints can be compared transparently.

### 2.2. Tropical Feedstocks as Materials Systems and Process Geographies

Tropical lignocellulosic residues differ from temperate wood not only in their composition but in their ash and silica burden, extractives, anatomical heterogeneity, moisture retention, storage stability, soil contamination, and the structure of their local supply chains [25,26,27,28,29,30,31]. These variables affect fibrillation energy, washing intensity, lignin accessibility, and the likelihood that lignin remains finely distributed rather than redepositing into coarse domains.

Table 1 compares the main tropical residue families through a process-geography lens. The goal is not to rank crops by cellulose content, but to identify where residue concentration, contamination, moisture history, and pretreatment compatibility make a lignin-containing nanocellulose route scientifically and industrially plausible. Representative studies span oil-palm, pineapple, banana, date-palm, and crop waste streams [32,33,34,35,36,37,38,39,40,41,42,43,44,45,46,47,48,49,50,51,52].

Oil-palm streams and sugarcane bagasse occupy the strongest near-term position because aggregation at mills favors wet preprocessing and integration with existing fractionation infrastructure [32,33,34,35,36,37,38,39,40,41,42,43,44,45,46,47,48,49,50,51,52]. Pineapple leaf fiber and banana pseudostem remain scientifically attractive, but their credibility depends more strongly on local stabilization, preprocessing, and contamination control [42,43,44,45,46]. Coconut husk and similarly diffuse residues should be treated as prospective cases unless an equally explicit process logic is demonstrated.

### 2.3. Evidence Logic: From Residue Management to Biorefinery Constraints

The strategic value of a tropical lignin–nanocellulose platform is clearest when framed as a biorefinery allocation problem rather than a nanofibril production problem in isolation [47,49,51,52]. The relevant question is not how to maximize purity alone, but how much multifunctionality can be preserved while minimizing bleaching, washing, drying, solvent recovery, and carbon loss across the process chain.

This framing makes process severity visible as a first-order design variable. Mild oxidation, hydrotropes, deep eutectic solvents, enzyme-assisted routes, steam explosion, and chemo-mechanical processing can all be useful, but only when the resulting interface/interphase survives dewatering, storage, and shaping without depending on laboratory-intensive dilution, freeze-drying, or solvent exchange [53,54,55,56,57,58,59]. Green extraction and lignin-retaining pretreatments are most convincing when solvent recyclability, water demand, corrosion, lignin condensation, and downstream solids management are considered together, rather than when a single extraction yield is reported in isolation.

**Figure 2:**
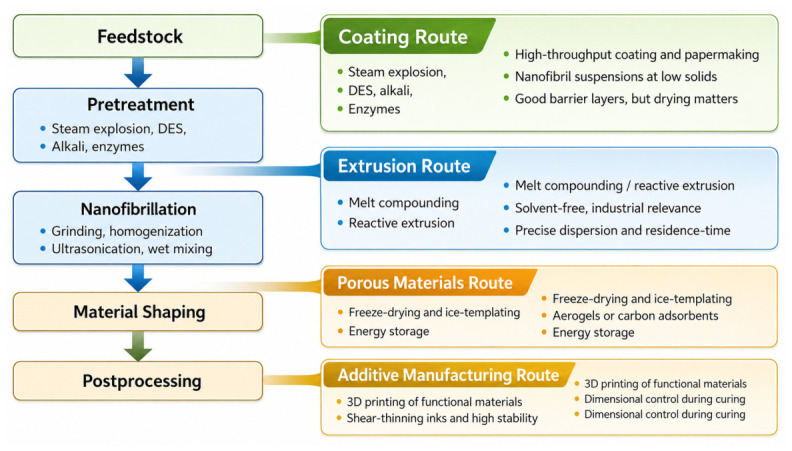
Decision pathway connecting process geography, pretreatment severity, lignin state, and application-specific shaping. Abbreviation: DESs, deep eutectic solvents.

Figure 2 translates this argument into a decision pathway. Hybrid performance is programmed long before compounding or film formation: feedstock conditioning shapes fractionation behavior, fractionation determines lignin state and fibrillation burden, and these two variables jointly constrain the type of interphase that can form later during coating, sheet formation, gelation, or melt processing.

### 2.4. Evidence Weighting and Comparative Synthesis Methodology

To avoid qualitative comparison alone, the review applies an explicit evidence-weighting scheme that separates robust, transferable observations from system-specific or process-dependent claims. The literature was categorized by (i) hybrid architecture class (residual lignin, redeposited lignin, lignin nanoparticles, reactive hybrids, and all-lignocellulosic structures), (ii) feedstock origin and process geography (mill-concentrated versus decentralized residues), and (iii) shaping route (coating, paper formation, porous structuring, and melt compounding). Each study was then scored using the reproducibility-oriented criteria summarized in Table 2.

- Tier I (high confidence; 8–10 points): Studies reporting controlled lignin state, quantitative nanocellulose morphology, defined processing history, application-relevant testing under humidity or realistic processing conditions, and appropriate controls or statistical replication.
- Tier II (moderate confidence; 5–7 points): Studies reporting composition and basic morphology but lacking at least one of the following: full interfacial characterization, humidity conditioning, processing traceability, or application-specific benchmarking.
- Tier III (low confidence; 0–4 points): Studies relying primarily on nominal lignin content or limited structural descriptors without process traceability, controls, or service-relevant testing.

Tier I corresponds to 8–10 points, Tier II to 5–7 points, and Tier III to 0–4 points. The aim is not to exclude lower-tier studies, but to prevent high-level design rules from being derived from evidence that lacks interfacial, process, or service-condition traceability.

The comparative conclusions and design rules in Section 5 and Section 6 are derived preferentially from Tier I evidence. Tier II and Tier III studies are used to identify trends and inconsistencies, but not to define universal composition-property thresholds. This framework is not a statistical meta-analysis; it is a conservative route to improve interpretability across heterogeneous lignocellulosic systems.

## 3. Distinct Hybrid Architectures and Interphase Logic

### 3.1. Chemical Complementarity Is Necessary, but Not Sufficient

The appeal of lignin–nanocellulose hybrids begins with their chemical complementarity. Cellulose provides a semicrystalline, high-aspect-ratio scaffold rich in hydroxyl functionality and network-forming potential [3,4,5,60,61,62]. Lignin contributes aromaticity, ultraviolet absorption, antioxidant capacity, surface energy modulation, and, in some cases, a lower apparent polarity than fully purified cellulose [10,11,15,16,17,18,19,63,64]. This complementarity explains why lignin retention can sometimes improve wetting in less-polar matrices while still preserving a fibrillar reinforcement network.

However, chemical complementarity alone does not fully address interphase compatibility. Lignin is structurally heterogeneous and can behave as a surface modifier, a colloidal phase, a brittle inclusion, a radical scavenger, or a processing contaminant depending on how it survives or reappears during pretreatment and drying. The correct question is therefore not whether lignin is generically beneficial, but whether a given lignin state strengthens or destabilizes the interface relevant to a specific shaping route and service environment.

### 3.2. Architecture Classes Should Not Be Merged Analytically

Lignin-containing nanocellulose should therefore be treated as a family of architecture classes rather than as an incompletely purified version of conventional nanocellulose [15,16,17,18,19]. At least five non-equivalent classes recur in the literature: residual-lignin CNFs/CNCs produced directly from partially delignified biomass; systems in which lignin redeposits or phase-separates during pretreatment, washing, drying, or compounding; deliberate assemblies of nanocellulose with technical lignin or lignin nanoparticles; compatibilized or carrier-assisted hybrids designed for thermoplastic transfer; and all-lignocellulosic sheets or molded structures in which lignin acts as an internal matrix or water-resistance phase.

These classes are not interchangeable because they exhibit different failure mechanisms and do not serve the same applications [65,66,67,68,69,70,71,72]. Residual-lignin fibrils may retain network continuity and moderate hydrophobic shielding. Redeposited lignin can introduce stress concentration, drainage penalties, or colloidal instability. Lignin nanoparticles can add multifunctional aromatic domains without entirely sacrificing the cellulose nano-network. Compatibilized thermoplastic hybrids remain highly sensitive to drying and melt-transfer history. All-lignocellulosic sheets follow another logic: their performance depends on how lignin redistributes during consolidation, drying, and rewetting.

**Figure 3:**
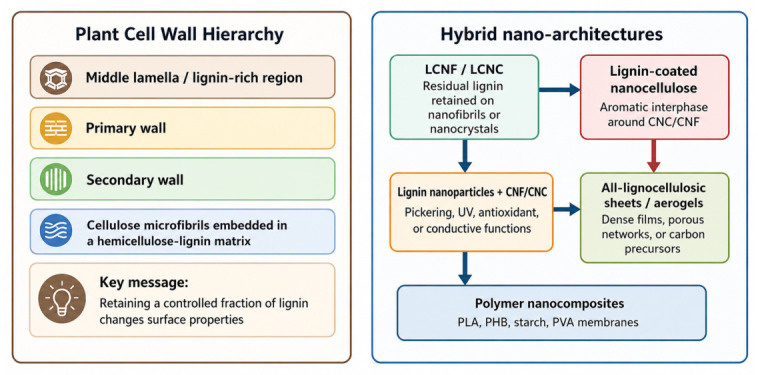
Distinct hybrid architectures considered in this review: residual-lignin nanofibrils or nanocrystals, redeposited-lignin systems, lignin nanoparticle assemblies, compatibilized thermoplastic hybrids, and all-lignocellulosic sheets. Abbreviations: CNFs, cellulose nanofibrils; CNCs, cellulose nanocrystals; LCNFs, lignin-containing cellulose nanofibrils; LCNCs, lignin-containing cellulose nanocrystals; PLA, poly(lactic acid); PHB, polyhydroxybutyrate; PVA, poly(vinyl alcohol).

Figure 3 can therefore be interpreted as a decision map rather than a purity hierarchy. The main analytical function of the scheme is to separate the amount of lignin from its state and to show that different interfaces must be evaluated against different failure modes.

The distinction is especially important for tropical residues because non-wood lignocellulose often carries extractives, ash, and anatomical heterogeneity that influence fractionation behavior and lignin relocation. A paper that reports only lignin percentage and a microscopy image does not yet demonstrate which interphase was created, nor whether that interphase could survive drying, storage, and shaping.

Based on the current evidence base, residual-lignin fibrils and all-lignocellulosic sheets are the most credible architectures for paper-like structures, coatings, and selected porous media. Lignin nanoparticle assemblies are credible when multifunctionality must be concentrated at a surface. Compatibilized thermoplastic hybrids remain case-dependent because interphase gains are easily lost during drying and melt processing [65,66,67,68,69,70,71,72,73,74,75,76,77,78].

The key consequence is straightforward: results should not be generalized directly across architecture classes. A positive outcome in a lignin nanoparticle coating, for example, does not validate a residual-lignin thermoplastic composite, and a strong all-lignocellulosic sheet does not by itself justify a biomedical claim.

This architecture split is the conceptual foundation of the present review because it converts ‘lignin-containing nanocellulose’ from a loose compositional label into an engineering family with distinct interphase logics. Taken together, these classes support the core claim of the review: bulk lignin fraction is a weak predictor of performance unless architecture class, spatial distribution, and shaping route are specified.

## 4. Interphase Engineering and Pretreatment Routes

### 4.1. Non-Equivalent Routes to Interphase Control

The interface is the decisive feature in almost every lignin–nanocellulose hybrid system. In hydrophilic matrices and dense paper-like networks, hydrogen bonding and capillary consolidation dominate. In less-polar matrices, a moderate lignin-rich surface or a compatibilized intermediate can improve wetting and stress transfer. In active surface systems, interphase design must also preserve access to lignin’s aromatic or phenolic functions. These are not variations in the same mechanism but rather different interphase strategies with distinct failure modes [69,70,71,72,77].

Four routes recur most often, but they should be compared not only by their limitations but also by the function they enable. Controlled retention of residual lignin is attractive when process simplification and wet processing are priorities. Colloidal co-assembly with technical lignin or lignin nanoparticles is attractive when UV shielding, antioxidant behavior, or Pickering stabilization must be concentrated at a surface. Reactive compatibilization is justified when permanent coupling is indispensable. Carrier-assisted transfer is mainly relevant when nanofibrils must survive drying and melt compounding [24,42,71,72,79,80].

Table 3 organizes these routes by interphase mechanism and dominant liability rather than by chemistry alone. That distinction matters because two formulations can use different chemistries yet still aim for the same interphase outcome, whereas apparently similar chemistries can fail differently when the lignin state differs.

Table 3 makes clear that the relevant comparison is not which chemistry appears most sophisticated, but which route achieves the required interphase with the lowest combined penalty in drying burden, humidity sensitivity, processing complexity, and loss of fibrillar continuity.

### 4.2. Pretreatment Severity, Fibrillation, and Failure Modes

Pretreatment determines not only fibrillation energy but also the chemical and spatial identity of the final interface. Mild oxidative routes, organosolv-type fractionation, hydrotropes, deep eutectic solvents, steam explosion, and chemo-mechanical strategies can all enable lignin-containing nanocellulose, yet they do so by changing lignin accessibility, cellulose disintegration behavior, and colloidal stability in different ways [47,48,49,50,51,52,53,54,55,56,57,58,59,82,83,84,85,86,92,93,94]. At the shaping stage, binding, coating, membrane, additive-manufacturing, and dispersion studies extend this process-history argument to final material forms [87,88,89,90,91,95,96,97]. Pretreatment should therefore be understood as interphase programming rather than as upstream purification alone.

A scale-up caveat follows immediately. Many laboratory-successful routes still rely on dilute suspensions, repeated washing, freeze-drying, or solvent exchange before compounding or coating. Those operations can erase the sustainability gains often claimed for lignin-containing systems and can also create the very lignin aggregation later attributed to material composition. A robust hybrid route is not merely one that gives a favorable microstructural morphology; it is one that preserves the intended interface/interphase through dewatering, drying, storage, and shaping.

**Figure 4:**
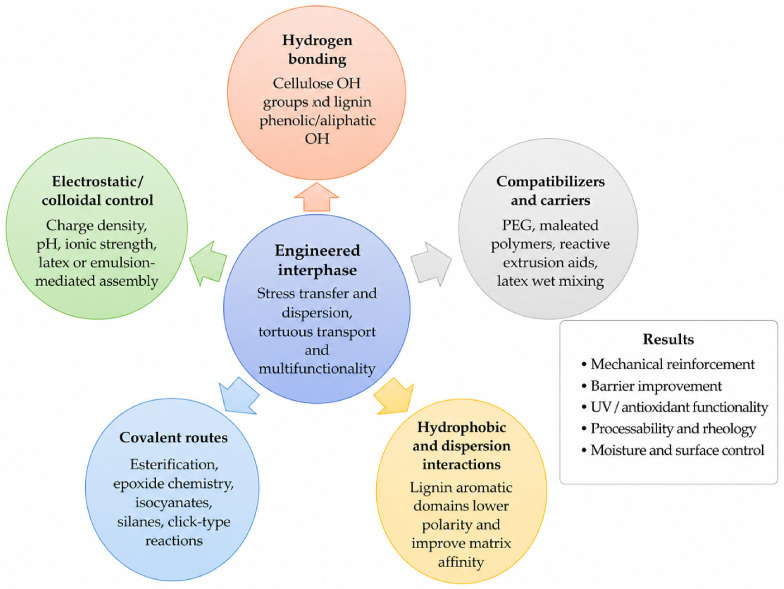
Interphase engineering routes, dominant liabilities, and representative failure modes in lignin-containing nanocellulose materials. Abbreviations: PEG, poly(ethylene glycol); OH, hydroxyl group.

Figure 4 condenses the main engineering routes and their associated failure modes. The point of the scheme is not to suggest that every hybrid needs all of these mechanisms at once. It is to show that interphase design is a multi-parameter problem in which hydrogen bonding, charge, covalent coupling, carrier effects, and process history interact.

Hydrogen bonding remains the most fundamental interparticle mechanism because cellulose offers abundant hydroxyl functionality, and lignin still contains phenolic and aliphatic hydroxyls. These interactions help form dense networks and tortuous pathways, but they are insufficient when the matrix is highly hydrophobic or when humidity weakens cohesion [96,97].

Charge and colloidal stabilization represent a second layer of design. Mild oxidation or charge tuning can improve fibrillation and suspension stability while preserving some lignin, but excessive charge or uncontrolled ionic conditions can also destabilize assembly or alter drainage and coating behavior.

Covalent strategies occupy a narrower but important window. Esterification, epoxide chemistry, silanes, isocyanates, and related reactions can reduce interfacial slippage and improve moisture resistance, yet they also add synthetic complexity and can alter the native colloidal behavior of nanocellulose. Their use is therefore best justified when the target matrix or service environment truly requires stronger or more permanent coupling.

Compatibilizers and carriers provide a more industrially pragmatic alternative for many thermoplastic systems. Poly(ethylene glycol), plasticizers, or selected dispersing routes can improve the transfer of lignin-containing fibrils into PLA- or PHB-like matrices, but these aids must ultimately be judged by their effects on moisture response, thermal history, and final interphase stability.

Viewed together, Figure 4 and Table 3 reinforce the main design lesson of this section: interphase engineering is not about maximizing the number of interactions; it is about choosing the minimum set of interactions that remains compatible with the shaping route, humidity exposure, and the target application.

## 5. Comparative Structure–Property Synthesis

### 5.1. Mechanical Response and Stress Transfer

Mechanical performance in lignin–nanocellulose hybrids depends on a coupled cascade of variables: fibril aspect ratio, dispersion state, porosity, interfacial continuity, and moisture content. Available studies indicate that residual lignin can improve mechanical response by enhancing wetting or preserving a connected fibrillar network, particularly in selected thermoplastic systems and dense sheet structures [69,70,88,98,99,100,101,102]. The benefit is real but conditional and architecture-dependent.

The same literature also explains many failures. When lignin persists as coarse domains, overplasticizes the interface, suppresses fibrillation, or breaks network continuity, tensile performance plateaus or declines. Any mechanical claim that omits humidity conditioning, solids history, or drying route should therefore be treated cautiously, because those variables can dominate the measured outcome as strongly as composition.

### 5.2. Barrier, Thermal, and Surface Performance

Lignin-rich interfaces often yield greater gains in thermal, barrier, and surface properties than in dry tensile strength. Aromatic domains can promote char formation, ultraviolet absorption, antioxidant activity, and modified wetting, while dense fibrillar networks provide the tortuous pathways needed for gas, oil, and grease barrier performance [85,88,89,90,91,103,104,105,106,107,108,109,110]. For packaging and coating systems, this combination is one of the strongest arguments for controlled lignin retention.

The trade-off is equally consistent. The color deepens, transparency decreases, and performance becomes more sensitive to lignin distribution and moisture history. These penalties are acceptable when the target product is a grease barrier, a UV-protective surface, a natural-looking, paper-like sheet, or an active coating. They are far less acceptable when optical clarity, extractable control, or strict color uniformity dominate the specification.

### 5.3. Rheology, Shaping, and Processability

Rheology is often the hidden determinant of whether laboratory properties can be translated into manufacturing. Nanocellulose suspensions are already strongly shear-thinning and can form gels at low solids; lignin retention can either improve or complicate this behavior depending on colloidal state, ionic environment, and solids history [99,111,112,113,114,115,116,117,118,119,120]. Coatings, papermaking, direct-ink writing, and thermoplastic compounding should therefore not be treated as equivalent outlets. Each imposes its own acceptable viscosity window, recovery behavior, and tolerance for drying-induced migration.

The evidence summarized in this section does not support universal pro- or anti-lignin claims. The credible design window is application-dependent. Moderate, well-distributed lignin often enhances UV shielding, antioxidant activity, oil resistance, and compatibility with less-polar phases, whereas high or poorly distributed lignin usually compromises transparency, fibril individualization, and reproducibility.

**Figure 5:**
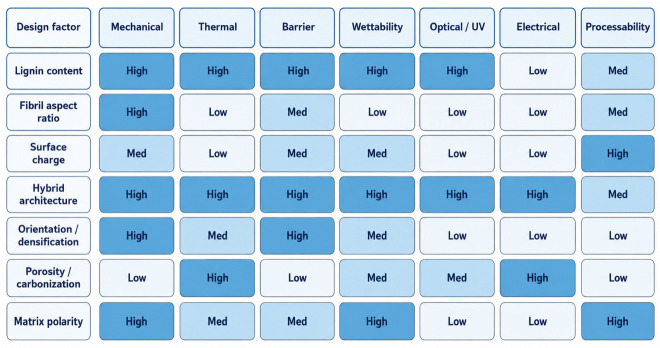
Confidence-ranked design map linking lignin state, nanocellulose morphology, process severity, and target properties. Abbreviation: UV, ultraviolet.

Figure 5 is best read as a comparative design map and confidence screen. Its function is to make trade-offs explicit and to show why the same structural feature can be beneficial in one product window and damaging in another. The message is not that lignin should always be retained, but that retention is useful only when the resulting interphase aligns with the target property, shaping route, and deployment scenario.

A strong review should therefore evaluate not only the property improvements but also the cost of obtaining them in terms of the pretreatment severity, drying burden, and moisture sensitivity. That is where interphase engineering becomes more than a materials description and becomes a design framework.

Apparent contradictions in the literature often arise because nominal lignin content is used as a proxy for architecture. Under the evidence-weighting framework introduced in Section 2.4, only studies that link lignin distribution, morphology, and process history can support transferable structure–property conclusions; the remaining literature is useful for trend detection but not for broad quantitative rules.

### 5.4. Representative Quantitative Observations and Normalization Limits

Quantitative comparisons are valuable only when they are interpreted within their architecture class and testing context. Table 4, therefore, lists representative experimental anchors rather than universal thresholds. The examples show that lignin-containing nanocellulose can deliver measurable gains in its wetting, oil resistance, oxygen barrier, thermal stability, and selected thermoplastic reinforcement; they also show that mechanical and rheological benefits are not automatic and can reverse when lignin distribution, humidity, morphology, or drying history are uncontrolled.

### 5.5. What the Current Evidence Can and Cannot Support

The present evidence base supports directionally robust design rules, but not universal quantitative thresholds. Too few studies simultaneously normalize lignin state, solids history, humidity conditioning, surface chemistry, and shaping route to justify claims such as an optimal lignin percentage across feedstocks or applications. Where quantitative comparisons appear in the literature, they usually remain architecture-specific and process-specific.

For that reason, this review intentionally ranks windows by confidence rather than by peak reported performance. A moderate, reproducible improvement under realistic processing should be prioritized over a large laboratory gain achieved after severe dilution, solvent exchange, or poorly reported drying history.

## 6. Confidence-Ranked Product Windows and Application-Backward Design Rules

### 6.1. Selected Thermoplastics: A Medium-Confidence Window

A wide range of polymer matrices have been combined with lignin and nanocellulose, or directly with lignin-containing nanocellulose, including PLA, PHB/PHA-like systems, starch-rich matrices, PVA, and selected model matrices used to isolate interfacial effects [122,123,124,125,126,127]. This literature is useful, but it also shows why thermoplastic translation should be treated as a medium-confidence rather than a default window. The drying, redispersion, carrier choice, residence time, and shear history often determine the outcome as strongly as the chemistry.

PLA remains the clearest benchmark because it is commercially visible, processable by casting, extrusion, and hot pressing, and sensitive to interfacial wetting. When moderate-lignin fibrils are transferred without coarse aggregation, their stiffness, barrier behavior, and sometimes toughness can improve; for example, PLA/9-LCNF films have shown higher tensile strength and modulus than pure PLA, whereas excessive lignin or poorly controlled compounding can reduce reproducibility [69,70]. PHB/PHA systems are attractive because their biodegradability aligns with lignocellulosic fillers, but brittleness, crystallization behavior, and thermal degradation during compounding remain limiting variables [102,128]. Starch- and PVA-rich matrices are more hydrophilic and can interact strongly with nanocellulose, but moisture sensitivity and plasticizer migration become central design constraints [127,129,130,131,132,133]. PBAT/PBS-like biodegradable polyesters and polyolefin blends remain less mature for LCNF transfer because compatibilization and drying history dominate performance. The defensible claim is therefore conditional: polymer composites are viable when the interface survives processing, not simply because lignin is present.

### 6.2. High-Confidence Windows: Packaging Layers, Papers, Coatings, and Porous Media

Surface-based platforms are the most credible near-term application space for tropical lignin–nanocellulose hybrids. Compared with bulk thermoplastics, papers, coatings, membranes, and selected porous media tolerate color more easily, operate at a lower solid throughput, and directly exploit functions enhanced by lignin retention, especially oil resistance, UV shielding, antioxidant activity, and selective wettability. Thin active layers are often more persuasive than thick self-standing structures because they concentrate functionality where mass transfer actually occurs.

### 6.3. Confidence-Ranked Design Rules and Application Matching

Application matching should set the required degree of purification. High-confidence windows are those in which lignin adds a function already aligned with the shaping route: grease/oil barrier papers, active or UV-shielding coatings, paper-like multilayers, and selected porous media. Medium-confidence windows include selected thermoplastics. Low-confidence or strongly conditional windows include biomedical interfaces, additive-manufacturing, and nano-reactor or energy-material concepts.

From that application-backward perspective, several rules become governing design criteria. Feedstocks should first be screened by process geography and contamination, not only by cellulose content. Lignin content is never a sufficient descriptor without lignin state. The shaping route must be chosen early because coating, papermaking, gel casting, and melt compounding reward different interfaces. Pretreatment must be interpreted as interphase programming because it fixes lignin accessibility, fibrillation behavior, and colloidal state [47,48,49,50,51,52,53,54,55,56,57,58,59,82,83,84,92,93,94,134,135,136,137].

Finally, application-relevant humidity, wetting, rheology, and process history must be incorporated into testing if performance claims are to be credible. The economically optimal route is therefore rarely the maximum-delignification route; it is the minimum-severity route that meets a defined specification under realistic service conditions.

The confidence hierarchy in Table 5 is a practical prioritization filter. It directs research and investment toward applications in which the lignin state can be controlled without incurring excessive processing penalties, especially in coatings, packaging layers, and porous media. It also keeps exploratory fields, such as biomedical interfaces and additive manufacturing, from being overinterpreted before extractables, rheology, sterilization, and long-term stability are demonstrated.

Table 5 converts these rules into confidence-ranked product windows. Table 6 reorders the evidence from the opposite direction and shows that the most transferable rule in the field is a distribution rule rather than a composition rule: finely distributed lignin is frequently useful; coarse or redeposited lignin is frequently destabilizing.

The practical value of Table 5 is that it prevents overextension of application claims: not every promising function justifies the same process burden. Surface-based windows tolerate color and exploit lignin more directly; thermoplastics demand far stricter control of drying, aggregation, and melt-transfer history.

Table 6 complements that application lens by showing that the strongest comparative tendency in the field is not a universal composition optimum but a spatial-distribution rule. Once lignin coalesces or migrates during drying, the interphase usually becomes less reliable, regardless of the nominal lignin percentage.

Taken together, Table 5, Table 6 and Table 7 define the central design logic of this review: Table 5 ranks product windows, Table 6 links lignin state to dominant outcomes, and Table 7 converts those tendencies into an architecture-level confidence screen.

### 6.4. Emerging Uses Should Be Judged by Interphase Realism, Not Novelty Alone

Emerging uses are scientifically valuable, but they should be treated as conditional until interphase realism, process continuity, and benchmarking are demonstrated alongside novelty.

One of the most instructive developments is the creation of water-stable lignocellulosic sheets and molded structures in which lignin functions as an internal matrix or wet-support phase rather than as a passive impurity [67]. This direction matters because it aligns a lignin-enabled mechanism with a plausible product logic for plastic-replacement sheets and molded products.

Biomedical and antimicrobial interfaces remain low-confidence opportunities rather than primary routes to deployment. Residual lignin introduces variability in color, extractables, sterilization response, and cytocompatibility, which sharply narrows the acceptable design window [142]. The scientifically honest position is not blanket enthusiasm or blanket dismissal, but strong qualification: these systems may be useful only when composition is tightly controlled and extractables, sterilization, and long-term response are explicitly tested.

For that reason, biomedical translation should not be used as the primary justification for lignin-containing systems. Packaging, coatings, paper-like structures, and selected porous media are currently the more convincing near-term outlets because they align better with the functions lignin actually adds and with the process simplifications lignin retention can realistically provide.

Lignin-containing nanocellulose is also attractive as a multifunctional nano-reactor or carbon precursor because aromatic motifs can participate in the in situ formation of nanoparticles and improve carbon yield during thermal conversion. These directions are valuable when they exploit lignin’s chemistry deliberately rather than simply inheriting it from insufficient purification.

Such work is especially interesting when lignin participates actively in redox chemistry, surface stabilization, or carbon-architecture control, as shown in studies on nanoparticle generation, wet-spun hydrogels, and carbon aerogels [68,73,74,75]. In confidence terms, however, these remain low-to-medium-confidence windows until process continuity, yield, and application benchmarking are demonstrated more systematically.

Additive manufacturing belongs to the same category of conditional promise. Printable pastes or inks must remain shear-thinning and self-supporting while drying without catastrophic cracking, binder migration, or collapse [73,95]. Here, again, interphase realism matters more than novelty: rheology, solids content, drying history, and postprocessing stability will decide whether the concept scales.

## 7. Process Realism, Deployment Scenarios, Circularity, and Research Agenda

The circular-bioeconomy promise of tropical lignin–nanocellulose is strongest when residues are abundant, under-valorized, and already connected to industrial infrastructure. Yet the decisive comparison is not ‘bio-based versus fossil’ in the abstract, but one tropical deployment scenario versus another [143,144,145,146,147,148,149].

Three scenarios occur: First, centralized wet routes connected to mills or processing hubs offer the strongest near-term case because residues can be stabilized and fractionated before uncontrolled drying. Second, decentralized residues can remain viable when they are preprocessed locally into wetter, cleaner, higher-value intermediates. Third, dry, diffuse collection routes are the least credible unless the target product has very high value density and can absorb the extra handling burden.

### 7.1. Deployment Scenarios, Sustainability Metrics, and Tropical Scale-Up

The available life-cycle and techno-economic literature repeatedly identifies pretreatment chemistry, washing intensity, fibrillation energy, drying burden, and solvent recovery as dominant hotspots in nanocellulose production [143,144,145,146]. For tropical systems, this means that moisture-preserving logistics, simplification of wet handling, and avoidance of unnecessary drying may matter more than marginal gains in purity.

**Figure 6:**
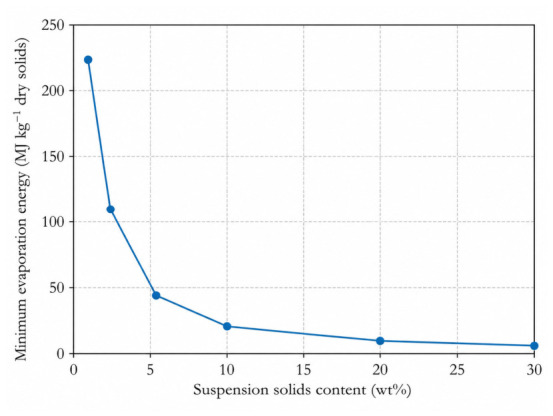
Author-calculated thermodynamic lower-bound energy required to evaporate water as a function of nanocellulose suspension solids content. The calculation assumes 1 kg of dry nanocellulose solids and uses $E_{min}=2.26[(1/C_{s})-1]$ MJ kg^−1^ dry solids, where $C_{s}$ is the suspension solid mass fraction and 2.26 MJ kg^−1^ is the latent heat of vaporization of water. The estimate excludes sensible heat, bound-water effects, equipment losses, heat recovery, and solvent-recovery penalties; therefore, real industrial drying demand is expected to be higher. Abbreviations: MJ, megajoule; $C_{s},solid$ mass fraction.

To make this drying burden explicit, Figure 6 presents an author-calculated thermodynamic lower-bound estimate for the energy required to evaporate water from nanocellulose suspensions as a function of solids content. The calculation assumes 1 kg of dry nanocellulose solids. For a suspension with a solid mass fraction, the total suspension mass required is $1/C_{s}$, and the corresponding water mass to be evaporated is $(1/C_{s})-1$. The theoretical minimum evaporation energy was therefore estimated as $E_{min}=2.26[(1/C_{s})-1]$ MJ kg^−1^ dry solids, using 2.26 MJ kg^−1^ as the latent heat of vaporization of water.

The magnitude is not trivial. At 2 wt% solids, complete water evaporation requires removing about 49 kg of water per kg of dry nanocellulose, corresponding to a theoretical latent-heat minimum of approximately 111 MJ kg^−1^ dry solids before equipment inefficiencies are included. At 10 wt% solids, the analogous minimum falls to approximately 20 MJ kg^−1^ dry solids. At 20 wt% and 30 wt% solids, the theoretical minima decrease further to approximately 9 and 5 MJ kg^−1^ dry solids, respectively. These values are not experimental drying-energy data; they are conservative thermodynamic baselines that exclude sensible heating, bound-water effects, dryer inefficiencies, heat recovery, solvent-recovery penalties, and equipment-specific operating conditions. Therefore, real industrial drying demand is expected to exceed the plotted values. The calculation nevertheless demonstrates why wet integration and high-solids processing are not minor engineering details but central scale-up criteria.

This drying-energy argument has direct implications for tropical lignin-containing nanocellulose platforms. Lignin-containing routes may outperform highly purified routes only when lignin retention reduces bleaching demand, washing intensity, dewatering burden, additive complexity, or drying energy across the process chain. If a lignin-containing route still requires repeated dilution, solvent exchange, freeze-drying, powder redispersion, or extensive post-treatment, its circularity claim becomes weak even when the final material shows promising laboratory properties.

**Figure 7:**
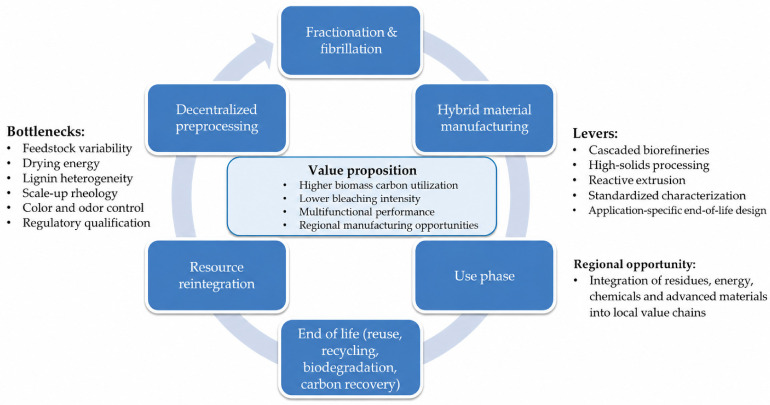
Scenario-dependent tropical biorefinery roadmap and deployment screen for lignin-containing nanocellulose platforms.

Figure 7 should therefore be interpreted as a scenario-dependent roadmap rather than as a guaranteed circular loop. This scenario-based logic is consistent with broader lignocellulosic biomass valorization frameworks, which emphasize that feedstock selection, process configuration, product portfolio, and value-chain integration jointly determine technical and economic feasibility [150]. At the process-chain level, the strongest candidate remains the integrated mill-centered route, particularly for oil-palm streams and sugarcane bagasse. These residues are already concentrated in industrial processing hubs, allowing wet preprocessing, fractionation, fibrillation, and direct conversion into coatings, paper-like layers, molded structures, or selected porous media. This route minimizes uncontrolled drying and improves the probability of preserving a reproducible lignin-containing interphase.

A second, conditional route is decentralized preprocessing, in which pineapple leaf fiber or banana pseudostem residues are washed, stabilized, and partially converted near the source into wet, cleaner, higher-value intermediates before long-distance transport. The weakest route is decentralized dry handling followed by rewetting and redispersion, because it combines a high logistics burden, a high drying-energy penalty, hornification risk, lignin redistribution, and poor interphase reproducibility.

This distinction matters because the same laboratory material can appear feasible or less feasible depending on the deployment scenario. A coating-grade lignin-containing nanofibril prepared from a wet mill stream is a very different proposition from a similar nominal nanofibril obtained after extensive drying, storage, and redispersion of diffuse residues.

Tropical strategy should therefore be written in terms of process geography and product windows, not crop names alone. Near-term deployment is most persuasive when wet intermediates can move directly into coatings, paper-like layers, molded structures, or selected porous media; it is less persuasive when the route depends on dry powders, multi-solvent transfer, or incompatible end-of-life claims.

Figure 7 is therefore most useful when read alongside the previous sections. Its purpose is to connect interface/interphase design to process-chain realism and to remind the reader that circularity claims must be critically evaluated with respect to water, energy, solvent recovery, logistics, and end-of-life coherence.

### 7.2. Critical Challenges and Standardization Priorities

Two further priorities deserve emphasis. First, future studies should report negative results and failure modes with the same scientific rigor as successful demonstrations, especially when lignin migration, color heterogeneity, drainage problems, or humidity sensitivity limit performance. Second, interfacial/interphase claims should increasingly be benchmarked against simplified, application-specific controls, e.g., purified CNF coatings, lignin-free paper barriers, or standard thermoplastic filler routes—so that the real value of lignin retention can be isolated rather than assumed.

Several bottlenecks still limit translation. Lignin heterogeneity remains under-characterized; studies often report lignin percentage without sufficient information on its molecular features, accessibility, condensation state, or spatial distribution. Structural reporting for nanocellulose is also inconsistent: fibril width, aspect ratio, colloidal stability, rheology, solids history, and moisture conditioning are not always quantified in a way that enables comparison across studies. Finally, much of the literature still demonstrates performance only after laboratory-intensive processing, which is difficult to justify for industrial-scale implementation.

The most productive future direction is application-specific interphase design. Rather than asking whether lignin is generally good or bad, the field should ask which lignin state, at which location, and in which morphology, enables a target function for a defined process route [15,16,19,23,54,55,56]. The recent acceleration of feedstock-specific reviews on pineapple-derived nanocellulose, date-palm nanocellulose, oil-palm nanocellulose for paper and packaging, and industrial-symbiosis routes for oil-palm residues confirms that generic ‘tropical biomass’ narratives are no longer sufficient [151,152,153,154].

Likewise, recent reviews on lignin for active food packaging, lignin nanoparticles, and lignin-based composites, together with new sugarcane-based studies on milder or one-step routes to lignin-containing nanofibrils, reinforce the same conclusion: performance improvements are achieved when aromatic functionality is placed at controlled interfaces while pretreatment, washing, and drying are simplified rather than intensified. Future studies will be more comparable when they report a minimum set of parameters linking feedstock chemistry, lignin state, fibril morphology, surface chemistry, moisture conditioning, and process history [155,156,157,158,159,160,161].

Table 8 condenses the minimum characterization framework and should also be read as an evidence-weighting tool. The goal is intentionally conservative. None of the requested descriptors is excessive; together they form the minimum evidence needed to distinguish a true interphase effect from artifacts introduced by uncontrolled fractionation, drying, or testing.

Used consistently, the reporting framework in Table 8 would move the field from descriptive comparisons toward more predictive engineering. That shift is essential if tropical lignin-containing nanocellulose is to become a serious materials platform rather than an endlessly variable family of promising case studies.

Green extraction technologies should be evaluated as integrated process options rather than as isolated chemistry choices. Hydrotropic fractionation, deep eutectic solvents, organosolv routes, enzyme-assisted treatments, nitro-oxidation, and steam explosion each produce distinct lignin states and solvent/water recovery burdens. Table 9 summarizes how these routes connect to economics, environmental performance, and digital optimization. Advanced AI/ML tools are not proposed as a substitute for experiments; rather, they can reduce experimental burden by screening feedstock variability, predicting delignification/fibrillation windows, ranking drying scenarios, and coupling property prediction with TEA/LCA constraints [150,161,162].

### 7.3. Comparative Process Scenarios

Scenario A is the mill-concentrated route: oil-palm residues and sugarcane bagasse can be fractionated while still wet, preserving lignin distribution and reducing drying/redispersion artifacts. This is the strongest scale-up pathway for coatings, paper-like layers, molded structures, and selected porous media.

Scenario B is decentralized preprocessing: pineapple leaf fiber and banana pseudostem can be viable when sorting, washing, stabilization, and partial conversion occur near the source. Without those controls, storage decay, ash/soil contamination, and lignin redistribution can erase interphase advantages.

Scenario C is the laboratory-intensive route: repeated washing, solvent exchange, freeze-drying, or powder redispersion can generate attractive specimens but weak process claims. Results from this route should be presented as mechanistic evidence unless yield, energy, solvent recovery, and humidity history are quantified.

### 7.4. Digital and AI-Assisted Research Agenda

A practical AI/ML agenda for this field should start with data discipline. The minimum descriptors in Table 8 can be treated as the feature schema for supervised learning: feedstock fraction, ash/extractives, lignin content and state, charge density, fibril width/aspect ratio, solids history, humidity conditioning, shaping route, and property outputs. With such descriptors, regression and classification models can predict whether a route is likely to yield a coating-grade suspension, a thermoplastic-compatible filler, or a porous precursor. Recent work on ML-guided lignocellulosic pretreatment shows that gradient-boosting-type models can achieve useful predictive performance for delignification and fractionation variables, but the same approach will only be reliable for LCNF composites if negative results and process failures are reported with the same care as successful demonstrations [161].

The most credible near-term use of AI is therefore not autonomous material discovery but decision support: selecting residue-to-product pathways, identifying low-severity pretreatment windows, ranking drying/transport scenarios, and coupling predicted performance with TEA/LCA objectives. This approach would allow tropical biorefineries to prioritize experiments that are both scientifically interpretable and industrially plausible, rather than optimizing peak laboratory properties detached from process geography.

## 8. Conclusions

This review supports a clear design principle: lignin-containing nanocellulose is best understood as an interphase engineering platform rather than as incompletely purified cellulose. In tropical biomass systems, performance is controlled more reliably by the lignin state, spatial distribution, fibril morphology, moisture history, and shaping route than by bulk lignin content.

The most defensible near-term applications are packaging layers, paper-like structures, coatings, and selected porous materials, in which controlled lignin distribution can provide UV shielding, antioxidant response, oil resistance, and wettability control without imposing prohibitive processing complexity. Thermoplastic composites remain viable but process-sensitive; biomedical, additive-manufacturing, and nano-reactor claims require stricter validation.

The field should now move from generic biomass substitution claims to evidence-weighted, application-backward design. Future studies should report feedstock identity, pretreatment severity, lignin state, nanocellulose morphology, surface chemistry, moisture conditioning, shaping history, and scale-up metrics. Under that discipline, tropical lignin-containing nanocellulose can become a credible industrial platform rather than a collection of promising yet difficult to compare case studies.

## Figures and Tables

**Table 1:** Tropical feedstock families are interpreted as materials systems and process geographies.

Feedstock	Tropical-Specific Constraint/Opportunity	Most Credible Process Logic	Near-Term Product Window/Refs.
Oil-palm residues	Centralized, lignin-rich streams and heterogeneous fractions, but have strong mill integration	Wet chemo-mechanical or mild selective pretreatment integrated at the mills	Packaging layers, paper-like sheets, renewable coatings [32,33,34,35,36,37,38,41]
Sugarcane bagasse	Centralized mill residue is favorable for wet handling and fractionation integration	Steam explosion, hydrotropes, or mild oxidative routes that preserve an interpretable lignin state	Lignonanocellulose films, coatings, and hybrid fibrils [47,49]
Pineapple leaf fiber	High-cellulose and has long fibers, but involves diffuse collection and contamination risk	Near-source preprocessing plus steam explosion or oxidative fibrillation	Lightweight sheets, functional papers, selected high-value composites [42,44]
Banana pseudostems/stems	Abundant and low-value, but have a high water content and decentralized logistics	Near-source stabilization and conversion into fibers or biopolymer intermediates	Packaging-oriented biocomposites and functional fibers [43,45,46]
Date-palm waste	Regionally strategic residue with strong reinforcement potential	TEMPO-assisted or mild lignin-retaining fibrillation	Reinforcing LCNF and region-specific composite uses [22]

**Table 2:** Operational evidence-weighting rubric used to assign Tier I–III confidence levels.

Criterion	0 Points	1 Point	2 Points
Lignin state and distribution	Only nominal lignin content reported	Lignin content plus indirect spectroscopy or microscopy	Lignin content plus spatial distribution or morphology resolved by microscopy/spectroscopy
Nanocellulose morphology	No fibril/crystal morphology reported	Representative images only	Width, length/aspect ratio, degree of fibrillation, and distribution descriptors were reported
Process traceability	Pretreatment/drying history unclear	Pretreatment is described, but the drying/solids history is incomplete	Pretreatment, washing, drying/never-dried state, solids, and shaping history specified
Application-relevant testing	Only generic characterization	One relevant test without humidity or service context	Property testing aligned with product window, including humidity, wetting, rheology, or processing conditions
Controls and reproducibility	No lignin-free or process control	Partial controls or single replicate/limited statistics	Appropriate controls, replicates/statistics, and explicit limitations

**Table 3:** Hybrid architecture classes, interphase mechanisms, and dominant liabilities in lignin-containing nanocellulose systems.

Strategy	Main Mechanism	Expected Advantage/Dominant Liability	Refs.
Residual-lignin retention	Partial hydrophobic shielding with preserved fibrillar connectivity	Process simplification and UV/antioxidant function/risk of suppressed fibrillation or color variability if lignin is coarse	[15,16,20,21,22,69,70]
Lignin nanoparticles with CNFs/CNCs	Colloidal co-assembly and surface-localized aromatic domains	Active surfaces, UV barrier, Pickering stabilization/risk of colloidal instability or aggregation	[13,23,65,66,68]
Mild oxidation or charge tuning	Adjusts fibrillation and colloidal stability while preserving part of the lignin	Better dispersion and tunable rheology/risk of charge-induced drainage or assembly penalties	[22,47,51,81,82,83,84]
Reactive compatibilization	Ester, epoxide, silane, or other covalent coupling	Stronger coupling and reduced leaching/risk of synthetic complexity and altered colloidal behavior	[42,72,79,80]
Carrier-assisted melt processing	Temporary plasticization or dispersion aid during compounding	Better filler transfer into thermoplastics/risk of lost fibrillar continuity during drying and melt history	[24,69,71,72]
Layer-by-layer or coating assembly	Sequential deposition and dense nano-network formation	Thin high-functionality layers with high barrier potential/risk of humidity-sensitive consolidation and coat-weight dependence	[85,86,87,88,89,90,91]

**Table 4:** Representative quantitative comparative observations used to anchor the evidence-weighted synthesis.

System/Architecture	Quantitative Observations	Main Property Message	Interpretive Limitation	Refs.
Residual-lignin nanopapers (0–14% lignin)	Water contact angle increased from ca. 35° to 78°; oxygen permeability decreased by up to 200-fold; tensile strength 116–164 MPa; modulus 10.5–14.3 GPa.	Surface and oxygen-barrier gains exceeded changes in dry tensile response.	Film density and humidity history remain decisive; lignin content alone is not predictive.	[20]
CNF vs. LCNF paper coatings, 16 g m^−2^ coat weight	CNF and LCNF coatings both reached kit No. 12; WVP ca. 5.0–5.3 g mm m^−2^ kPa^−1^ day^−1^; WCA 59.4° for CNF and 82.2° for LCNF; OTR increased strongly at 90% RH.	LCNFs exhibited improved surface hydrophobicity and prolonged oil-holding behavior while retaining water-vapor barrier properties similar to those of CNFs.	The oxygen barrier remained moisture-sensitive, showing that hydrophobicity does not eliminate humidity effects.	[88]
PLA/LCNF thermoplastic films	Pure PLA: ca. 35.7 MPa and 1.8 GPa; PLA/9-LCNF: ca. 48.9 MPa and 2.9 GPa; Tg decreased from 61.2 °C to 52.6 °C in PLA/14-LCNF.	Moderate lignin improved PLA compatibility and stiffness/strength; excessive lignin shifted thermal-mechanical behavior.	Data are matrix-, dispersion-, and processing-specific; powder/redispersion routes can erase gains.	[70]
Cationic/enzymatic LCNF films	LCNF films reached ca. 50 MPa and 2.5 GPa; enzymatic films showed WVTR 144–153 g m^−2^ day^−1^ versus 418–440 g m^−2^ day-1 for cationic films; enzymatic LCNF had OTR below 2 cm^3^ m^−2^ day^−1^.	Pretreatment chemistry can dominate barrier and mechanical responses, even when lignin retention is similar.	Direct comparison requires density, charge, and conditioning normalization.	[121]
LCNF suspension rheology from wood and soybean hulls	Wood-derived CNF suspensions showed approximately ten-fold higher viscosity than soybean-derived CNFs; all samples were shear-thinning and primarily elastic, with tan delta around 0.1.	Rheology depends on morphology, charge, raw material, and associated hemicellulose/pectin/lignin, not only on lignin percentage.	Rheological values are concentration- and protocol-dependent; reporting solids and shear history is mandatory.	[120]

**Table 5:** Application-backward design rules with confidence-ranked product windows for packaging, coatings, porous media, and thermoplastic matrices.

Application	Recommended Hybrid Architecture	Primary Design Objective	Key Processing Caution/Refs.
Grease/oil barrier paper, board, and multilayer papers (high confidence)	Residual-lignin CNF coatings; dense LCNF multilayers	Lower surface energy while preserving dense fibrillar tortuosity and coatability	Control coat weight, drying, humidity response, and rewetting [85,88,89,90,91,104,105,106]
PLA/PHA films and molded bioplastics (medium confidence)	Moderate-lignin LCNFs; carrier-assisted transfer into thermoplastics	Improve wetting and stress transfer without losing fibril reinforcement	Prevent lignin agglomeration and color heterogeneity during melt processing [24,69,70,71,72,98,99]
Selected porous sorbents and oil/water separation media (high confidence)	LCNF aerogels; selectively hydrophobized lignin-rich porous networks	Tune wettability, capillarity, and selective sorption	Avoid pore collapse and poor wet resilience during drying/rewetting [85,108,109,110,113,114,115]
Active or UV-shielding packaging and surface coatings (high confidence)	Surface-lignin-rich CNFs or CNFs combined with lignin nanoparticles	Exploit UV absorption and antioxidant functionality at the surface	Balance opacity/color with migration, appearance, and humidity requirements [12,13,66,80,88,100,138]
Carbon precursors, printed structures, and energy materials (low–medium confidence)	Wet-spun lignin/CNF hydrogels; ice-templated lignin-cellulose aerogels	Maximize carbon yield while preserving hierarchical precursor structure	Manage shrinkage, solids loading, and porosity evolution during thermal conversion [73,74,75]
Biomedical or biointerface materials (low confidence)	Purified or tightly characterized low-extractable LCNF formulations	Use surface area and antioxidant behavior without uncontrolled extractables	Require cytocompatibility, extractables, sterilization, and long-term validation [65,79,101,139,140,141,142]

**Table 6:** Comparative evidence matrix linking lignin states to likely property outcomes and dominant failure modes.

Lignin State/Distribution	Typical Processing Context	Likely Interfacial Effect	Frequent Property Outcome	Refs.
Finely distributed residual lignin on fibril surfaces	Mild chemo-mechanical, organosolv, or hydrotropic fractionation followed by fibrillation	Lower apparent surface polarity with preserved nanofibrillar connectivity	Usually beneficial for UV screening, antioxidant function, selective wetting, and some less-polar compatibility; strongest evidence in coatings, papers, and selected thermoplastics	[15,16,17,18,19,21,22,81]
Coarse redeposited or phase-separated lignin domains	Insufficient fractionation control, lignin migration during drying, or poorly redispersed powders	Interphase discontinuity, stress concentration, and non-uniform wetting	Often detrimental: lower tensile reliability, heterogeneous color, unstable rheology, and poor film quality	[15,16,17,18,19,24,69,70,71,72]
Lignin nanoparticles combined with CNFs/CNCs	Colloidal co-assembly, coating routes, and Pickering-type formulations	Surface-localized aromatic multifunctionality without losing the cellulose nano-network	Credible route to active surfaces, UV barrier, emulsification, and carbon precursors when colloidal compatibility is maintained	[13,23,65,66,67,68,73,74,75]
Covalently compatibilized or carrier-assisted thermoplastic hybrids	Reactive compounding, surface coupling, or PEG-assisted transfer into thermoplastics	Stronger filler transfer and more stable interphase during melt processing	Potentially better stress transfer and moisture resistance, but benefits are route-sensitive and can disappear during processing	[24,42,71,72,79,80,98,99]
Strongly delignified nanocellulose networks	Severe bleaching and oxidation routes aimed at high purity	Maximized hydrogen-bonded cellulose network and optical clarity	Best transparency and dense barrier networks, but loss of lignin-enabled UV/antioxidant functions and some process-simplification advantages	[3,4,5,7,60,61,88,89,90,91,139]

**Table 7:** Evidence-weighted comparison of lignin-containing nanocellulose architectures across applications.

Architecture	Typical Lignin State	Best-Fit Applications	Main Advantage	Main Limitation	Evidence Strength
Residual-lignin CNFs	Finely distributed	Coatings, paper, packaging	Process simplicity, UV function, oil resistance	Moisture sensitivity and color	High (multiple consistent studies)
Redeposited-lignin systems	Heterogeneous, coarse domains	Limited	No robust advantage consistently demonstrated	Poor interphase continuity and instability	Low (contradictory evidence)
Lignin nanoparticles + CNFs	Controlled colloidal phase	Active surfaces, coatings, Pickering systems	Functional interface design	Colloidal-stability constraints	Moderate–high
Reactive hybrids	Covalently modified interfaces	Thermoplastics	Stronger adhesion and reduced slippage	Process complexity	Moderate
All-lignocellulosic sheets	Distributed matrix-like lignin	Molded materials, water-stable sheets	Wet stability and plastic-replacement potential	Structural variability	Moderate

**Table 8:** Minimum reporting and evidence-weighting set required for interpretable interphase claims and scalable processing arguments.

Parameter to Report or Define	Why It Matters	Minimum Recommended Evidence	If Omitted, the Main Interpretive Risk	Refs.
Feedstock identity, residue fraction, and pretreatment severity	Defines ash content, residual-lignin chemistry, accessibility, and lot-to-lot variability	Residue fraction, origin, season or lot, solids, time–temperature history, and reagent loading	Results cannot be transferred reliably across species or process routes	[32,33,34,35,36,37,38,41,42,43,44,45,46,53,54,55,56,57,58,59,82,83,84,92,93,94]
Lignin content and lignin state	Content alone does not explain performance unless distribution and chemistry are known	Acid-insoluble/soluble lignin plus spectroscopic or microscopic evidence of spatial distribution	False attribution of effects to lignin percentage alone	[15,16,17,18,19,21,22,81]
Fibril or crystal morphology	Controls percolation, network formation, and stress transfer	Width, length, or aspect ratio and representative microscopy with distribution descriptors	Reinforcement claims cannot be compared across studies	[3,4,5,15,16,17,18,19,60,61]
Surface chemistry, charge, and wetting	Governs dispersion, coating behavior, and colloidal stability	Zeta potential or charge density, FTIR/XPS or equivalent, and wetting descriptor when relevant	The origin of compatibility or instability remains speculative	[22,60,61,81,96,97]
Moisture state and conditioning	Strongly affects tensile, barrier, and dimensional data	Relative humidity conditioning before testing, water content if possible, and testing environment	Property gains are easily overstated or become irreproducible	[25,26,27,28,29,30,31,88,89,90,91,142]
Composite or coating processing window	Shear and thermal history can damage the interphase during scale-up	Solids content, drying route, temperature profile, residence time, and consolidation conditions	Property losses may be misassigned to chemistry rather than processing	[24,42,71,72,98,99,143,144,145,146]
Application window, deployment scenario, and sustainability metrics	Circularity requires more than renewable content and depends on where the material enters the value chain	Intended product window, centralized or decentralized route, water use, drying burden, solvent recovery, mass yield, and at least a TEA/LCA note when scale-up is claimed	Claims of scalability remain rhetorical instead of evidence-based	[76,143,144,145,146,147,148,149]

**Table 9:** Green extraction, scale-up, and AI/ML opportunities for tropical lignin-containing nanocellulose platforms.

Route or Digital Tool	Main Benefit	Scale-Up Constraint	Best Tropical Fit	Decision Metric
Hydrotropic or recyclable organic-acid fractionation	Atmospheric or mild fractionation with potential acid recovery	Acid recovery, corrosion, and lignin redeposition control	Sugarcane bagasse; mill-integrated residues	Mass yield, acid recycling, lignin distribution
Deep eutectic solvents (DESs)	Selective lignin extraction and tunable solvent design	Viscosity, solvent recovery, water dilution, and impurity accumulation	Oil-palm and bagasse fractions, where the solvent loop can be centralized	Solvent loss, delignification, and fibrillation energy
Steam explosion/hydrothermal pretreatment	Chemical-light opening of the cell-wall structure	Inhibitor formation, fiber damage, lignin relocation	Pineapple leaf fiber, banana pseudostem, sugarcane bagasse	Severity factor, fiber quality, wet stabilization
Enzyme-assisted or mild oxidative pretreatment	Lower chemical intensity and better surface-charge control	Enzyme cost, residence time, and sensitivity to contamination	Near-source preprocessing of clean residues	Charge density, rheology, yield, contamination tolerance
AI/ML and active-learning workflows	Predict pretreatment–property windows and reduce experimental matrix size	Requires curated datasets with standardized descriptors	All residue families once the feedstock/process metadata are reported	Cross-validated prediction error, TEA/LCA objective function

## Data Availability

No new data were created in this review. The study synthesizes the published literature cited in the reference list.

## Abbreviations

The following abbreviations are used in this manuscript:

CNFs	Cellulose Nanofibrils
CNC	Cellulose Nanocrystals
LCNF	Lignin-Containing Cellulose Nanofibrils
LCNCs	Lignin-Containing Cellulose Nanocrystals
PLA	Poly(lactic acid)
PHB	Polyhydroxybutyrate
PHAs	Polyhydroxyalkanoates
PVA	Poly(vinyl al-cohol)
DESs	Deep Eutectic Solvents
TEA	Techno-Economic Analysis
LCA	Life-Cycle Assessment
ML	Machine Learning
